# Random walk: Random number generation during backward and forward walking- the role of aging

**DOI:** 10.3389/fnagi.2022.888979

**Published:** 2022-09-28

**Authors:** Maxim Shapiro, Samuel Shaki, Uri Gottlieb, Shmuel Springer

**Affiliations:** ^1^The Neuromuscular and Human Performance Laboratory, Department of Physical Therapy, Faculty of Health Sciences, Ariel University, Ariel, Israel; ^2^Department of Behavioral Sciences, Ariel University, Ariel, Israel

**Keywords:** gait, aging, cognitive flexibility, gait variability, embodiment

## Abstract

Deficits in executive function, visuospatial abilities, and cognitive embodiment may impair gait performance. This study aimed to investigate the effect of age on random number generation (RNG) performance during forward and backward locomotion to assess cognitive flexibility and cognitive embodiment during walking. Another aim was to examine the effect of age on the associations of RNG performance during walking with stride time variability (STV), the percentage of double support (DS%), and visuospatial abilities as measured by a spatial orientation test (SOT). Twenty old (age 68.8 ± 5.3, 65% female) and 20 young (age 25.2 ± 2.2, 45% female) adults generated random numbers during backward walking (BW) and forward walking (FW) over-ground and over a treadmill with an internal focus of attention and visual-attentive distraction; six walking conditions in total. To assess cognitive flexibility, sample entropy was calculated for each RNG sequence. The average of the first 5 numbers in each RNG task was calculated to assess the relationship between small/large numbers and movement direction. STV and DS% were recorded using inertial measurement units, and spatial orientation was measured using a computerized test. The older subjects had less flexibility in generating random numbers in three of the six walking conditions. A negative correlation between RNG flexibility and STV was found in older adults during treadmill BW with visual-attentive distraction and forward over-ground walking, whereas no correlations were demonstrated in the young group. The spatial orientation score (a higher value means a worse outcome) correlated positively with RNG flexibility in the older group under all walking conditions, suggesting that older adults with better visuospatial orientation have lower cognitive flexibility, and vice versa. There was no correlation between small/large numbers and direction of motion in either group. The correlation between RNG flexibility and STV may indicate similar executive control of verbal and gait rhythmicity in old adults. Conversely, our results suggest that cognitive flexibility and visuospatial ability may decline differently.

## Introduction

For many years, walking was considered an automated activity controlled by central pattern generators at the spinal level, requiring minimal cognitive resources (Guertin, 2009). Over the past two decades, studies of cognitive function and gait have demonstrated a substantial interaction between cognitive domains and the control of walking. Evidence suggests that deficits in executive function, visuospatial processing, and memory resources might be the cause of movement impairments and gait disorders (Yogev-Seligmann et al., 2008; Kearney et al., 2013; Martin et al., 2013). It is well established that cognitive involvement in walking increases with age. Studies examining walking under demanding conditions, such as walking while performing two tasks simultaneously, have shown greater declines in motor and cognitive performance in the elderly compared with young adults (Brustio et al., 2017).

Declines in executive function, even in “healthy” aging, can lead to deterioration in gait performance due to decreasing cognitive reserves (Li et al., 2018). Moreover, the frontal-visuospatial network may regulate gait in the context of motor adaptation to changes in external visual stimuli during walking (Amboni et al., 2013). In addition, the visuospatial subcomponents of working memory may further strain executive function, as there is a negative linear correlation between age and working memory function (Shaw et al., 2006).

The embodied cognition perspective is an emerging area of research in cognitive sciences. This approach to cognition holds that our minds and thoughts are not separate from the environment, but that cognition involves and depends on the interaction between the brain, body, and external stimuli (Anderson, 2003). According to this theory, cognition is rooted in the sensorimotor system, which plays a role in both action implementation and conceptual processing. For example, words referring to motor abstracts have been shown to activate frontal and parietal motor areas (Harpaintner et al., 2020). Thus, the same neurons may be responsible for the conceptual representation of an action and its execution, suggesting a link between action and perceptual cognition. Declines in sensory, motor, and cognitive functions may affect embodiment during aging (Vallet, 2015). The degree of embodiment during aging may be assessed by the mental representation of abstract concepts and the relationship between action and perception (Costello and Bloesch, 2017).

An example of embodiment is the mental representation of numbers (Fischer and Shaki, 2018). A left-right orientation of a mental number line (MNL) has been demonstrated in many studies (Fischer and Shaki, 2014, 2018). When young adults were asked to generate random numbers during a lateral turn, they generated smaller numbers on average in the left turn than in the right turn, suggesting a spatial-numerical association (Loetscher et al., 2008; Shaki and Fischer, 2014). Although the presence of a MNL on the left-right axis is well established, numbers are also presented from back to front in everyday movement sequences (e.g., distance measurements in walking). However, the relationship between back-to-front walking and number representation has not been studied in detail (Winter et al., 2015).

The random number generation (RNG) task can be used to study both embodied cognition and executive function (Oomens et al., 2015). Previous literature shows that a decrease in executive function may be associated with serialization and repetition in the RNG task, resulting in less “randomness” (Peters et al., 2007). When comparing young and older subjects, the latter group produces fewer random sequences, supporting the notion that RNG is a cognitively demanding task that may decline with age (Van der Linden et al., 1998). In addition, young adults produce fewer random outcomes when paired with a second movement task (Dirnberger and Jahanshahi, 2010).

Backward walking (BW) is an additional method for assessing gait control (Taulbee et al., 2021). Compared to forward walking (FW), BW is a complex motor task with increased activation of cognitive and sensorimotor resources due to altered or absent visual feedback (Błażkiewicz, 2013). BW can be a novel task even for healthy individuals, as shown by Kurz et al. (2012), who found an increase in sensorimotor cortical activation, as measured by functional near-infrared spectroscopy, and greater stride-time variability during BW in healthy adults. This could imply that BW is a demanding task that requires increased cognitive resources.

The objective of the present study was to test the effect of age on RNG task performance during forward and backward locomotion, this can help to understand the differences in embodiment between young and old adults. Based on the claim that spatial abilities, working memory, and spatial representation of numbers share the same neural mechanism (Hawes and Ansari, 2020), another aim was to investigate the relationships between RNG flexibility during walking, to gait control, and visuospatial abilities. We hypothesized that: (1) A negative correlation between the degree of RNG flexibility during walking and gait control, as measured by stride time variability (STV) and percentage of double limb support (DS%), will be demonstrated in older adults but not in young adults. These spatiotemporal gait parameters are commonly used to assess gait control in the elderly (Hollman et al., 2011). Increased STV and prolonged DS% may indicate a less stable gait that requires more cognitive engagement (Al-Yahya et al., 2011; LaRoche et al., 2014), and a relationship between cognitive flexibility and gait outcomes has been previously demonstrated (Hobert et al., 2017). (2) Because both visuospatial ability and RNG may be mediators of working memory function (Baddeley et al., 1998; Wang et al., 2018), a negative correlation between visuospatial orientation, measured by angular error in identifying direction to objects, and RNG flexibility will be demonstrated in both groups. (3) Forward-backward MNL will be demonstrated during walking only in young adults: i.e., during the performance of the RNG task, young adults will generate on average larger numbers during FW compared to BW. This will not be observed in older adults, as there is evidence that older adults are less embodied (Costello and Bloesch, 2017).

## Materials and methods

### Participants

We used G*Power (version 3.1) (Faul et al., 2007) to determine the sample size with the following parameters: Power = 80, α = 0.05, and an effect size (*d* = 0.84) found in a previous study (Van der Linden et al., 1998) examining the effects of age on RNG flexibility when performing a parallel task (RNG index-young adults 0.08 ± 0.03, older adults 0.12 ± 0.06; the closer to 1, the lower the flexibility). This indicated that a sample size of 19 participants in each age group would be required to achieve our main objective, of testing the effect of age on RNG flexibility during FW and BW. Therefore, the study included a convenience sample of 20 older adults and 20 young adults. Young adults were included if they were between 18 and 30 years of age, not taking medications, and reported good general health. Older adults between the ages of 60 and 80 participated in the study if they lived in the community, were independent in activities of daily living, could walk without assistance, and had at least basic computer skills. Subjects with neurological, orthopedic, or visual impairments (e.g., age-related macular degeneration, glaucoma, cataract, diabetic retinopathy) or other comorbidities that could affect gait were excluded. Furthermore, to ensure that the participants had no underlying physical, behavioral, or mental health conditions, subjects in both groups had to score above 42 on the mental component and above 50 on the physical component of the Short-Form 12 Health Survey (SF-12) (Ware et al., 1995; Wee et al., 2008). In addition, elder participants were excluded if they scored less than 24 on the Montreal Cognitive Assessment (MoCA) (Carson et al., 2018). The study was approved by Ariel University Ethics Committee (approval number AU-HEA-SS-20200909). All subjects gave written informed consent to participate in the study.

### Procedure

Each subject participated in a single session that lasted approximately 60 min. Before assessing gait, the visuospatial ability was quantified using a computerized test of spatial orientation (SOT), which included a timed 12-task that measured 2-dimensional mental rotation ability (Hegarty and Waller, 2004; Friedman et al., 2020). On each of the 12 trials, participants were shown an array of objects and asked to imagine that they were at one object and facing a second object (an orientation object). They were asked to indicate the direction to a third object (the target object) by drawing a line from the center of the circle in the direction believed to be correct. The SOT was scored according to angular errors (a higher score indicates lower visuospatial ability) and averaged across all items for each participant.

Gait was assessed during the performance of an RNG task while walking forward and backward in randomized order under three conditions: (1) Walking over-ground (GRD)—in this condition, subjects were instructed to walk at their comfortable pace along a 20-m in an obstacle-free hallway. (2) Walking on a treadmill with an internal focus of attention (TRD-F)—under this condition participants were asked to walk while focusing on the movement of their legs. (3) Walking on a treadmill with visual-attentional distraction (TRD-D)—subjects were instructed to focus on a red marker located in front of them. The marker was placed 2 m away from participants and approximately 30 cm above eye level. Participants were asked to identify the marker and it was verified that they could see it clearly before the treadmill tests began. Gait was assessed under different attentional scenarios, as attentional states have been shown to influence balance, movement automation, and walking efficiency in older adults (Kal et al., 2013; Mak et al., 2019). Before data collection, subjects were given the opportunity to become accustomed to walking on a treadmill, and gait speed was gradually increased for both FW and BW. The treadmill familiarization period lasted up to 6 min in each direction, to ensure safe ambulation by the subject while keeping minimal habituation to the testing condition (Wass et al., 2005; Meyer et al., 2019). The maximum comfortable walking speed achieved by each subject was used for all successive treadmill trails. For additional safety, subjects were harnessed while walking on the treadmill while the investigator stood next to the treadmill. The investigator walked behind the subjects during the GRD walking conditions.

To test RNG flexibility, subjects were given the same instructions before they began each walk: “While walking, say any sequence of numbers between 1 and 30 that comes to mind. Try to keep the sequence random.” Subjects were asked to say random numbers for as long as it took them to complete a 20-m over-ground walk or the distance they walked in 20 s on the treadmill. Subjects were asked to name the numbers as fluently as possible without receiving specific instructions on the rhythm of number generation (Jokar and Mikaili, 2012). The sequence of numbers was recorded using a commercially available voice recorder.

To assess the randomness of the RNG sequence, sample entropy (SampEn) was calculated for each RNG sequence (Delgado-Bonal and Marshak, 2019). SampEn is a mathematical algorithm created to measure the repeatability within a series, which can provide an improved evaluation of its regularity (Yentes et al., 2013). Lower values of SampEn reflect less flexible and more predictable sequences and vice versa. To assess the presence of MNL on the back-to-front axis, the average of the first 5 numbers in each RNG task was calculated. The use of the first 5 numbers was intended to capture the content of working memory at the time of movement initiation (Pöppel, 1997).

Overground walking speed was measured with a stopwatch over a marked distance of 20 m, and gait velocity on the treadmill was measured with the Treadmill. STV and DS% of gait cycle were measured during all walking trials. STV is a marker of rhythmicity and stability of gait and is calculated as follows (Hausdorff, 2005):


(1) $$S\,T\,V=\frac{\sigma }{\mu }*100$$

Where σ is the standard deviation of stride times and μ is the average stride time. The DS% describes the ratio between the amount of time both feet touch the ground and the duration of the entire gait cycle. Higher values of DS% indicate a less stable, more demanding gait in the elderly (Prince et al., 1997). A wireless IMU system (Delsys Trigno, Delsys Inc., Boston, MA, USA) sampling at 370 Hz was used to record acceleration data from lightweight, rectangular IMUs (dimensions: 37 × 26 × 15 mm, weight < 15 g). The sensors were attached to the lateral side of the heel using a double-sided adhesive interface (Delsys Inc., Boston, MA, USA). Data were recorded using EMGworks acquisition software (version 4.7.8, Delsys, Boston, MA, USA) and exported to Python v3.7 for further processing to analyze the spatiotemporal gait outcomes. The peaks of sagittal angular velocity and acceleration data were identified as initial foot contact (IC) and terminal foot contact (TC) during both FW and BW. In a previous work, we demonstrated excellent reliability (interclass correlation > 0.9) between this method and data acquired with an eight-camera motion capture system (Qualisys, Göteborg, Sweden) and analyzed with Visual 3-D software (C-motion, Inc., Kingston, ON, Canada) to identify IC and TC (Gottlieb et al., 2020).

### Statistical analysis

Demographic variables were compared between groups using Student’s *t*-tests. A 2 × 2 × 3 Linear mixed-effects model (LMM) was applied to evaluate the effects of group, walking direction (FW, BW), and condition (GRD, TRD-F, TRD-D) on RNG sequence SampEn (i.e., flexibility). *Post hoc* pairwise *t*-tests were used to compare significant effects as appropriate. LMMs were also used to assess the effects of group, walking direction, and walking condition on gait outcomes (STV and DS%), while controlling for gait speed. Between-group comparisons were performed for each walking direction and condition separately by calculating additional six LMMs (with walking speed as a covariate for gait outcomes). Holm correction for multiple comparisons was applied for all *post-hoc* tests. The LMMs’ residuals were inspected through histograms and quantile-quantile plots to verify normal distributions. Partial η^2^ effect sizes (η^2^ = 0.01 indicates a small effect; η^2^ = 0.06 indicates a medium effect; η^2^ = 0.14 indicates a large effect) were calculated for each model, and Cohen’s *d* effect sizes (*d* = 0.2 indicates a small effect; *d* = 0.5 indicates a medium effect; *d* = 0.8 indicates a large effect) were calculated for *post hoc* comparisons (Lakens, 2013). To better understand the relationships between RNG flexibility, gait control parameters (STV, DS%), and visuospatial orientation (SOT score), separate Spearman’s correlations were calculated for each group in each gait condition. Correlations were interpreted according to the following scale: weak < 0.4, moderate 0.4–0.7, and strong > 0.7 (Akoglu, 2018).

Finally, to assess the existence of a MNL, an additional LLM was performed to determine the effects of group, walking direction, and walking condition on the average of the first 5 numbers in the RNG task. Statistical analysis was performed in R v.4.0.3, with a significance level set at *p* < 0.05.

## Results

### Basic characteristics

Table 1 provides an overview of the basic characteristics of the participants. The mean age of the older group (65% female) was 68.8 ± 5.3 years, and that of the young group (45% female) was 25.2 ± 2.2 years. A significant difference between groups was found in the physical component of the SF −12 (53.1 ± 5.1 vs. 56.3 ± 1.6, *p* = 0.013). The mean MoCA score for the old group was 26.8 ± 1.7, and only three subjects scored a minimum of 24 points. Compared to the young group, older adults had lower gait speed during treadmill walking (FW and BW, *p* < 0.001) and during BW/GRD (*p* = 0.001), but not during FW/GRD (1.16 ± 0.22 vs. 1.21 ± 0.19, *p* = 0.428). Older adults had significantly lower spatial orientation ability (higher SOT score) compared to young adults (68.5 ± 32.3 vs. 26.3 ± 18.5, *p* < 0.001).

**TABLE 1:** Participants’ basic characteristics.

	Old	Young	*P*-value
Age	68.8 ± 5.3	25.2 ± 2.2	**<0.001**
Height (cm)	163.4 ± 8.0	168.8 ± 11.5	0.091
Body mass (kg)	70.7 ± 16.0	68.2 ± 12.9	0.592
BMI (kg/m^2^)	26.4 ± 5.2	23.8 ± 3.0	0.068
SF-12			
PCS (pts)	53.1 ± 5.1	56.3 ± 1.6	**0.013**
MCS (pts)	55.2 ± 8.1	54.9 ± 3.7	0.853
MoCA score (pts)	26.8 ± 1.7		
Treadmill FW velocity (m/s)	0.65 ± 0.25	1.12 ± 0.27	**<0.001**
Treadmill BW velocity (m/s)	0.32 ± 0.11	0.53 ± 0.14	**<0.001**
Over-ground FW velocity (m/s)	1.16 ± 0.22	1.21 ± 0.19	0.428
Over-ground BW velocity (m/s)	0.74 ± 0.2	0.94 ± 0.16	**=0.001**
SOT score	68.5 ± 32.3	26.3 ± 18.5	**<0.001**

BMI, Body Mass Index; MCS, Mental Component Score; PCS, Physical Component Score; MoCa, Montreal Cognitive Assessment; FW, Forward walking; BW, Backward walking; SOT, Spatial Orientation Test. The bold values indicate significant p value (p < 0.05).

### Random number generation flexibility

The results of the RNG sequence SampEn are presented in Table 2. The LMM performed to determine the effects of group, walking direction, and walking condition on the RNG sequence SampEn revealed significant effect for group [X^2^_(1)_ = 11.3, *p* = 0.001, partial η^2^ = 0.21], but not for walking direction [X^2^_(1)_ = 0.2, *p* = 0.657, partial η^2^ = 0.01] or condition [X^2^_(2)_ = 0.1, *p* = 0.947, partial η^2^ = 0.06]. No significant interaction effects were found. The between-groups pairwise comparisons of the RNG sequence SampEn values in each walking condition are also described in Table 2. The older group demonstrated significantly lower RNG flexibility in the following conditions: FW/GRD (*p* = 0.040), FW/TRD-D (*p* = 0.048), and BW/TRD-F (*p* = 0.035). A trend for lower RNG flexibility in the older group was also found in the other conditions: FW/TRD-F (*p* = 0.068), BW/GRD (*p* = 0.056), and BW/TRD-D (*p* = 0.056).

**TABLE 2:** The RNG sequence SampEn values and between-group comparisons.

	Old	Young	*P*-value	Cohen’s *d*
**FW**				
- GRD	0.20 ± 0.16	0.39 ± 0.26	**0.040**	−0.89
- TRD-F	0.18 ± 0.12	0.25 ± 0.11	0.068	−0.59
- TRD-D	0.18 ± 0.16	0.31 ± 0.16	**0.048**	−0.83
**BW**				
- GRD	0.18 ± 0.12	0.29 ± 0.17	0.056	−0.77
- TRD-F	0.18 ± 0.12	0.30 ± 0.13	**0.035**	−0.92
- TRD-D	0.18 ± 0.11	0.26 ± 0.10	0.056	−0.75

RNG, Random Number Generation; SampEn, Sample Entropy; FW, Forward Walking; BW, Backward Walking; GRD, Overground; TRD-F, Treadmill with attentional focus; TRD-D, Treadmill with attentional visual distraction. Values are presented as means ± SD. Holm correction for multiple comparisons were used to calculate p-values. The bold values indicate significant p value (p < 0.05).

### Gait control outcomes

The LMM for STV revealed significant effects for group [X^2^_(1)_ = 10.7, *p* = 0.001, partial η^2^ = 0.32], direction [X^2^_(1)_ = 23.2, *p* < 0.001, partial η^2^ = 0.64], but not for condition [X^2^_(2)_ = 5.3, *p* = 0.069, partial η^2^ = 0.11]. None of the interaction effects were significant. According to *post-hoc* tests, older adults had significantly higher levels of STV in FW/GRD (*p* = 0.006, Cohen’s *d* = 1.04), FW/TRD-D (*p* = 0.04, Cohen’s *d* = 0.81), and BW/TRD-D (*p* = 0.006, Cohen’s *d* = 1.02).

The LMM for the DS% revealed significant effects for direction [X^2^_(1)_ = 43.9, *p* < 0.001, partial η^2^ = 0.58], but not for group [X^2^_(1)_ = 0.1, *p* = 0.734, partial η^2^ = 0.01] or condition [X^2^_(2)_ = 3.2, *p* = 0.204, partial η^2^ = 0.07]. None of the interaction effects were significant. There was no between groups significant differences in DS% in either of the walking directions or conditions. Results of *post hoc* LMMs for STV and DS% are shown in Table 3.

**TABLE 3:** *Post hoc* linear-mixed models’ results for comparing between groups stride time variability and double support % while controlling for walking speed, in each direction and condition.

		STV			DS%		
	Adj. speed	Old	Young	*P*-value	Old	Young	*P*-value
**FW**							
- GRD	1.19	3.2 (2.8–3.7)	2.3 (1.9–2.7)	**0.006**	26.0 (22.6–39.3)	23.5 (20.2–26.8)	1.00
- TRD-F	0.88	3.0 (2.4–3.6)	2.4 (1.8–3.1)	0.228	28.7 (25.0–32.4)	30.6 (26.8–34.3)	1.00
- TRD-D	0.88	3.5 (2.9–4.0)	2.4 (1.8–2.9)	**0.040**	29.2 (25.2–33.1)	30.1 (26.1–34.1)	1.00
**BW**							
- GRD	0.84	5.6 (4.8–6.4)	4.5 (3.7–5.4)	0.141	40.2 (38.4–42.1)	36.9 (35.0–38.7)	0.072
- TRD-F	0.42	5.6 (4.7–6.5)	4.0 (3.2–4.9)	0.069	49.6 (47.2–52.0)	48.2 (45.8–50.6)	1.00
- TRD-D	0.42	6.0 (5.3–6.7)	4.3 (3.6–5.0)	**0.006**	52.2 (49.2–55.3)	48.8 (45.7–51.9)	0.694

STV, Stride Time Variability; DS%, Double Support Percentage; FW, Forward Walking; BW, Backward Walking; GRD, Overground; TRD-F, Treadmill with attentional focus; TRD-D, Treadmill with attentional visual distraction. Values are presented as estimated means (95% CI) after adjusting for walking speed (Adj. speed). Holm correction for multiple comparisons were used to calculate p-values. The bold values indicate significant p value (p < 0.05).

### Association between random number generation flexibility and stride time variability

**FIGURE 1:**
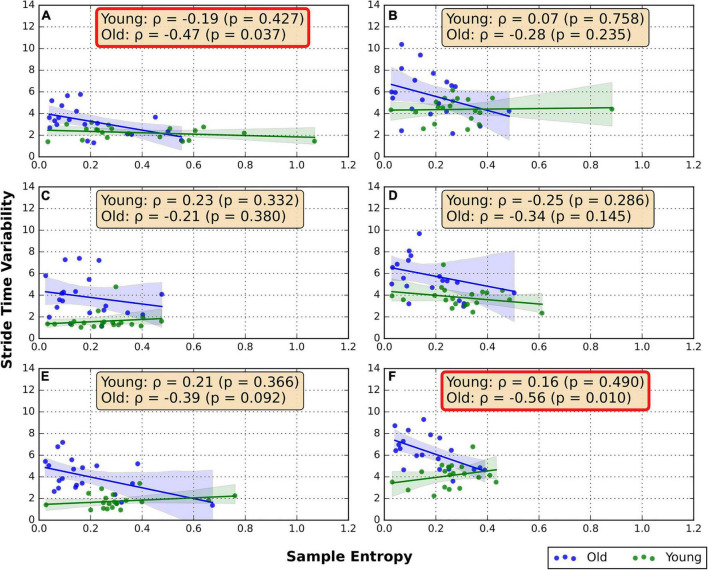
Spearman correlation between RNG flexibility and STV. **(A)** Overground forward walking, **(B)** overground backward walking, **(C)** treadmill forward walking with an internal focus of attention, **(D)** treadmill backward walking with an internal focus of attention, **(E)** treadmill forward walking with visual-attentional distraction, **(F)** treadmill backward walking with visual-attentional distraction. The red box indicates a significant correlation.

The correlations between the RNG sequence SampEn and STV are shown in Figure 1. In the old group moderate negative correlations were found between STV and SampEn in FW/GRD (ρ = −0.47, *p* = 0.04) and BW/TRD-D (ρ = −0.56, *p* = 0.01). In addition, a trend of weak negative correlation was found in the FW/TRD-D (ρ = −0.39, *p* = 0.09). No significant correlations were found in the young group (Figure 1).

### Associations between random number generation flexibility and double support percentage

**FIGURE 2:**
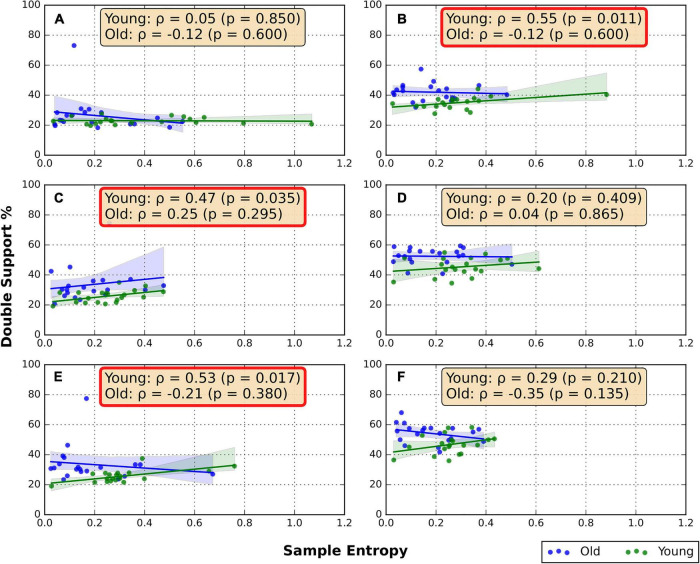
Spearman correlation between RNG flexibility and DS%. **(A)** Overground forward walking, **(B)** overground backward walking, **(C)** treadmill forward walking with an internal focus of attention, **(D)** treadmill backward walking with an internal focus of attention, **(E)** treadmill forward walking with visual-attentional distraction, **(F)** treadmill backward walking with visual-attentional distraction. The red box indicates a significant correlation.

The correlations between the RNG sequence SampEn and DS% are shown in Figure 2. Moderate positive correlations were found between DS% and SampEn in the BW/GRD (ρ = 0.55, *p* = 0.011), FW/TRD-F (ρ = 0.47, *p* = 0.035), and FW/TRD-D (ρ = 0.53, *p* = 0.017) conditions only in the young group. No significant correlations were found in the older group (Figure 2).

### Associations between random number generation flexibility and spatial orientation

**FIGURE 3:**
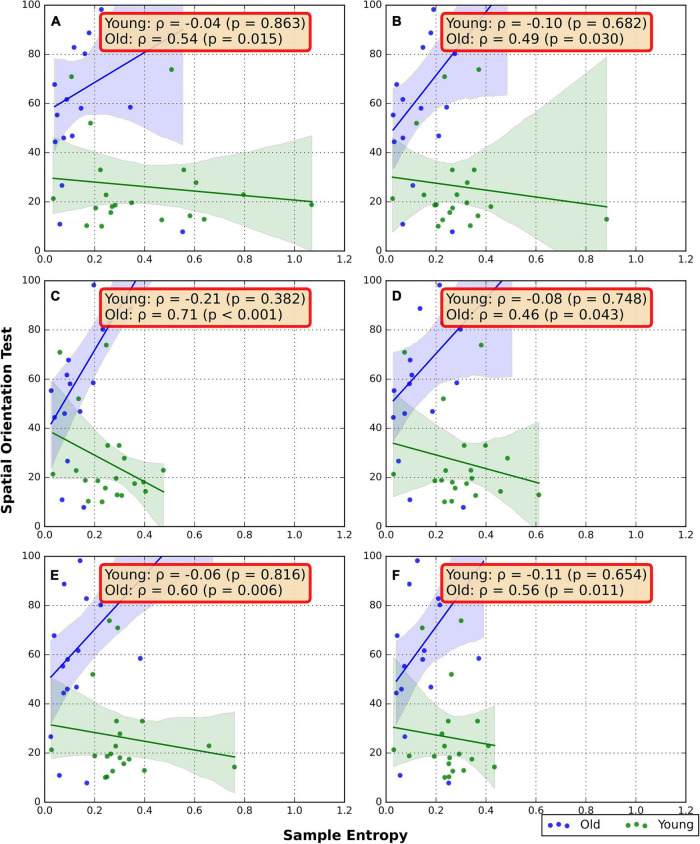
Spearman Correlation between RNG flexibility and SOT. **(A)** Overground forward walking, **(B)** overground backward walking, **(C)** treadmill forward walking with an internal focus of attention, **(D)** treadmill backward walking with an internal focus of attention, **(E)** treadmill forward walking with visual-attentional distraction, **(F)** treadmill backward walking with visual-attentional distraction. The red box indicates a significant correlation.

The associations between the RNG sequence SampEn and SOT are presented in Figure 3. In the older group, a strong positive correlation was found between RNG SampEn and SOT in the FW/TRD-F condition (ρ = 0.71, *p* < 0.001). Moderate positive correlations were also found in FW/GRD (ρ = 0.54, *p* = 0.015), BW/GRD (ρ = 0.49, *p* = 0.03), BW/TRD-F (ρ = 0.46, *p* = 0.043), FW/TRD-D (ρ = 0.6, *p* = 0.006), and BW/TRD-D (ρ = 0.56, *p* = 0.011). No significant correlations were found in the young group (Figure 3).

### The back-to-front mental number line

Table 4 presents the average of the first five numbers in each RNG task in both groups under all tested walking directions and conditions. The model which determined the effects of group, walking direction, and walking condition on the average of the first five numbers in the RNG task demonstrated a significant effect for group [X^2^_(1)_ = 4.4, *p* = 0.036, partial η^2^ = 0.08], walking condition [X^2^_(2)_ = 13.1, *p* = 0.001, partial η^2^ = 0.02] and group × walking condition interaction [X^2^_(2)_ = 12.2, *p* = 0.002, partial η^2^ = 0.06], but not for waking direction [X^2^_(1)_ = 0.7, *p* = 0.414, partial η^2^ < 0.01].

**TABLE 4:** Average of the first five numbers in each RNG task.

	Old		Young	
	FW	BW	FW	BW
- GRD	8.6 ± 4.7	9.3 ± 5.4	11.9 ± 4.3	11.7 ± 4.4
- TRD-F	12.2 ± 5.3	10.6 ± 6.3	11.8 ± 5.2	11.1 ± 4.0
- TRD-D	9.3 ± 6.0	8.7 ± 5.1	13.3 ± 4.8	12.1 ± 3.6

RNG, Random Number Generation; FW, Forward Walking; BW, Backward Walking; GRD, Overground; TRD-F, Treadmill with attentional focus; TRD-D, Treadmill with attentional visual distraction.

## Discussion

This study aimed to examine the effects of age on RNG task performance during forward and backward ambulation, as this could characterize both cognitive embodiment and executive function. Our findings showed that elderly individuals exhibited reduced flexibility in generating random numbers during FW and BW compared with young adults in three of the six conditions tested, while the rest of the conditions exhibited a similar although not significant trend. This may indicate a decline in executive abilities and is consistent with the work of Van der Linden et al. (1998), who reported differences between young and elderly subjects in generating random strings of letters. However, while Van der Linden et al. (1998) investigated the effects of age on a random generation task in a stationary position, the present study is the first to show such results during ambulation.

To examine the association between ambulation and cognition, we tested the correlation between gait control parameters (i.e., STV and DS%) to indicators of cognitive function (i.e., RNG flexibility and spatial rotation ability). A negative correlation between RNG flexibility and gait variability (i.e., increased STV) was found in older adults during backward treadmill walking with visual-attentional distraction, and during natural forward overground ambulation, while no correlations were demonstrated in the other walking conditions or the young group. This finding is consistent with previous evidence showing that gait control is associated with cognitive flexibility among older adults, and may suggest similar executive control of gait and RNG performance (Ble et al., 2005; Deshpande et al., 2009; Killane et al., 2014). However, here we extend these previous findings as none of the former studies reported an association between STV, an independent marker of gait rhythmicity and steadiness (Frenkel-Toledo et al., 2005), to cognitive flexibility. Previous reports have also shown that cognitive flexibility is likely associated with gait, particularly during complex walking conditions (Hirota et al., 2010; Killane et al., 2014; Hobert et al., 2017). In our study, it is reflected by the association between RNG flexibility and gait variability while walking backward in the TRD-D condition. Focusing on a single point in space while walking backward (as the subjects were instructed during this walking condition) limits the visual information, thus requiring more executive involvement in gait control. This is further supported by the results indicating only a trend between gait variability and RNG flexibility during a condition with more visual feedback such the FW/TRD-D. Yet, a correlation between gait variability and RNG flexibility was also demonstrated, in the old group, under natural forward overground walking. Future research that will investigate the association between cognitive flexibility and gait variability is warranted.

Our results uncovered a positive correlation between RNG flexibility and DS%, in the young group, in some of the testing conditions. Compared to older adults, young adults are not as dependent on visual information while walking and less frequently look to the ground (as instructed in the TRD-F condition) (Anderson et al., 1998). It is likely that the unfamiliar gait, in which the young subjects were tasked to walk while visually focusing on the movement of the lower extremities, caused the increase in the DS%. It is also possible that young healthy adults prioritized the cognitive task during treadmill walking, causing cognitive-motor interference and increased DS% (Small et al., 2021). In contrast to our hypothesis, a correlation between RNG flexibility and DS% was not evident in the old group. The average age of the older group in our study was 68.8 ± 5.3 years, a previous work showing that DS% does not increase during dual-task in healthy individuals in their 60 s may explain this result (LaRoche et al., 2014). Furthermore, while our subjects were not diagnosed with cognitive impairments, an increase in DS% during dual-task may be more relevant to subjects with mild cognitive impairment (Nascimbeni et al., 2015).

The aforementioned findings may suggest the possibility that STV is more sensitive to changes in executive control of gait than DS% in healthy older adults. In addition, the concept of rhythmic synchronization of neurophysiological activity may be the underlying mechanism for the association between STV and RNG flexibility, as both walking and generating numbers are rhythmic tasks (Farmer, 1998). This mechanism was previously observed in a study showing that backward counting may alter STV (Beauchet et al., 2010). It is recommended for future studies to compare the effect of different forms of verbal number generation (e.g., forward, backward, random) on the variability of gait.

Contrary to our assumption that the SOT score would be negatively associated with cognitive flexibility in both groups, our results uncovered positive significant correlations, in the old group, between visuospatial orientation score (a higher value means a worse outcome) and RNG flexibility under all walking conditions. These results may suggest that older individuals with better visuospatial orientation (i.e., lower SOT score) have lower cognitive flexibility (i.e., less randomness during the RNG task) and vice versa. Although both verbal and visuospatial working memory decline with age, there is evidence that these cognitive components may decline differently (Shaw et al., 2006; Kumar and Priyadarshi, 2013). Some of our elderly subjects who have a declined working memory and executive function may have relatively preserved visuospatial abilities. Our results may also suggest that RNG flexibility during walking is not solely related to cognitive domains of memory, and it may represent different aspects of cognitive function. It is also possible that older adults with higher visuospatial orientation have a more rooted representation of numbers that are not anchored in the backward to forward direction. Therefore, they prefer to generate consecutive numbers resulting in a lower RNG SampEn. It is important to note that this proposed explanation requires further investigation.

Per the concept of cognitive embodiment and the mental representation of numbers, we hypothesized the existence of a backward-forward MNL among the young group and not in the older group, indicating less cognitive embodiment. The results did not validate the existence of a backward-forward MNL in both groups. Previous studies have proven the existence of a MNL, embodied on the left-to-right axis (Shaki et al., 2009; Shaki and Fischer, 2014), suggesting that both words and numbers contribute to spatial representation, due to the specific direction of reading language and numbers (with a reversed association between left-to-right and right-to-left readers). Although several aspects of daily life ambulation include representations of numbers from back to front (e.g., measurements of distance during walking). These representations may not be sufficient to induce cognitive saturation that will establish an MNL. Further studies may consider measuring whether other conceptual abstracts are presented on a back-to-front axis.

The difference in RNG flexibility between groups in the present study may be in contrast to the MoCA score of the older subjects. Theoretically, one would not expect deficits in executive function, because the group of older adults had a mean MoCA score of 26.8 ± 1.7 (lowest score: 24, in 3 subjects only), indicating no major cognitive deficits. One possible explanation is that the MoCA assessment is not sensitive enough, because only 5 of the 30 items are directly related to working memory (Nasreddine et al., 2005). In addition, several studies have shown that cognitive assessment while walking may be more sensitive to cognitive decline (Springer et al., 2006; Srygley et al., 2009; Perrochon and Kemoun, 2014). Although cognitive load in RNG is likely to be lower than in other types of cognitive tasks (e.g., serial subtraction), it appears that reduced flexibility in the RNG task during walking is sensitive enough to detect deficits in executive function in healthy older adults.

Although the results of the present study do not have an immediate practical impact on the treatment of patients, they may provide clues for future treatment strategies. It could be suggested that elderly people with unstable gait be trained to count sequential (i.e., non-random) numbers aloud while walking forward and backward. There is evidence that counting aloud may have a regulating effect (comparable to a metronome) on individuals with irregular walking rhythms (Beauchet et al., 2010). It may also be beneficial to train subjects with good visuospatial and motor skills to generate random numbers aloud while walking to provide them a challenge and improve their performance. The beneficial effects of these treatment strategies should be confirmed in future studies.

The findings of this study should be interpreted with some limitations. Our protocol did not include an evaluation of RNG flexibility without ambulation or a walking trial without RNG task. Further research may include a comparison of RNG performance with and without movement and a baseline assessment of walking. Adequate spatial orientation abilities are essential for ambulation. To raise ecological validity, it is also recommended that future studies will incorporate a dynamic and more challenging assessment of spatial orientation during walking. Another methodological limitation of the present study may be related to the number of strides obtained to measure stride-to-stride variability and the sequence of numbers under each walking condition used for the SampEn calculation. The number of strides and the sequence of numbers collected in our study was approximately 30. Riva et al. (2014) claimed that at least 127 strides are required to quantify gait variability. In contrast, Kroneberg et al. (2019) indicated that gait variability assessment can be reliably performed with less than 15 strides. Similarly, although there is no consensus on the minimum sample for calculating entropy, it appears that SampEn can be reliable for short data sets (Yentes et al., 2013). Finally, a safety harness was used when walking on the treadmill but not during overground walking, which may affect the results. However, it has been shown previously that non-weight-supporting harness do not alter gait dynamics (Stout et al., 2016). Though we do not believe that these limitations had a major effect on our results, our findings should encourage further research on the associations between cognitive flexibility and the control of gait.

## Conclusion

Consisted with the paradigm of rhythmic synchronization of neurophysiological activity, we demonstrated for the first-time association between random numbers generation flexibility and gait variability, suggesting similar executive control of verbal and gait rhythmicity in old adults. Conversely, our results indicate that cognitive flexibility and visuospatial ability may decline differently. Obtaining more understanding of cortical gait control during aging may provide information that would allow designing interventions targeting specific brain mechanisms, to ensure better and healthy aging.

## Data availability statement

The raw data supporting the conclusions of this article will be made available by the authors, without undue reservation.

## Ethics statement

The studies involving human participants were reviewed and approved by the Ariel University Ethical Committee. The patients/participants provided their written informed consent to participate in this study.

## Author contributions

MS designed the study, performed data collection, and drafted the manuscript. SSh and SSp designed the study and performed critical review of the manuscript. UG performed data interpretation, statistical analysis, and review of the manuscript. All authors contributed to the article and approved the submitted version.
